# Hormonal and Subjectively Perceived Stress of the Emergency Physicians of the Airborne Rescue Service

**DOI:** 10.1007/s10880-021-09767-3

**Published:** 2021-02-17

**Authors:** Desiree Braun, Lorenz Theiler, Elmar Brähler, Katja Petrowski

**Affiliations:** 1 Department of Psychology and Psychotherapy, University Witten/Herdecke, Alfred-Herrhausen-Straße 50, 58455 Witten, Germany; 2 Medical Psychology and Medical Sociology, Clinic and Policlinic for Psychosomatic Medicine and Psychotherapy, University Medicine Mainz, Mainz, Germany; 3 Department of Anaesthesiology and Pain Therapy, Inselspital, Bern, Switzerland; 4 Department of Psychosomatic Medicine and Psychotherapy, University Medical Center Mainz, Johannes Gutenberg University Mainz, Mainz, Germany

**Keywords:** Stress, Cortisol, Emergency physician, Air rescue

## Abstract

Due to their work activities, emergency physicians are regularly exposed to exceptional mental and physical situations. In order to prevent stress-related illnesses, the triggers of hormonal and subjectively perceived stress must be understood better. On a sample of emergency physicians from two air rescue services (*N* = 80), the cortisol awakening response (CAR) was determined on flight rescue days, clinic days, and days off. Pearson correlations showed significant connections between the CAR on flight rescue days and individual scales of the Trier Inventory for Chronic Stress (TICS) and the Perceived Stress Scale (PSS). The results indicate that a lower subjective stress level is associated with higher levels of hormonal stress. Stepwise regression analyses showed a significant influence of the number of professional years, subjectively perceived stress, pressure to succeed, and social isolation. The results suggest that the hormonal stress burden of emergency physicians is in a complex relationship with perceived strain.

## Introduction

Stress response is a physical adaptation to stressful life events. It deals with threatening situations by activating the adaptive homeostatic systems of the body, which should enable an appropriate handling of the stressor (Chrousos, 2009). When people are exposed to stress-inducing situations too often or for too long, stress can become chronic and lead to mental and physical illnesses (Chrousos, 2009; Herbert, 2013; Brown, Varhese, & McEwen, 2004).

McEwen’s theoretical model of allostasis and allostatic load (McEwen, & Lasley, 2002) describes that stress response is beneficial to maintaining the organism’s stability. In the event of too severe or prolonged exposure to a stressor, stress response will become dysfunctional (no recovery or inappropriate stress response) and, thus detrimental to bodily health.

At the physiological level, when confronted with a threatening stimulus, the hypothalamic-pituitary-adrenocortical axis (HPA axis; Pariante, & Lightman, 2008) is activated. Initially, the adrenocorticotropic releasing hormone (CRH) is released from the hypothalamus and the adrenocorticotropic hormone (ACTH) from the pituitary gland. As a result, cortisol is released from the adrenal cortex. The concentration of cortisol follows a typical course. Within a few minutes, the cortisone level rises sharply until it reaches its maximum after approx. 30 min and then drops off again continuously (Pariante, & Lightman, 2008). The extent of the cortisol reaction thus serves as a suitable measure for the quantification of hormonal stress. However, chronic stress can change the cortisol response permanently. Due to a characteristic rise of cortisol after awakening, which is influenced by long-term stress, cortisol awakening response (CAR) is a typical marker for measuring chronic stress (Fries, Dettenborn & Kirschbaum, 2009; Wüst, Federenko, Hellhammer, & Kirschbaum, 2000; O’Connor, Walker, Hendrickx, Talbot, & Schaefer, 2013; Inslicht et al., 2012). In twins, it could be shown that an increase in CAR is related to increased subjective stress (Wüst et al., 2000) as well as stress-related thinking (O’Connor et al., 2013). Police officers with increased CAR are at increased risk of developing peritraumatic dissociation or an acute stress disorder (Inslicht et al., 2012).

Certain occupational groups are particularly at risk as they are regularly exposed to stress-related situations and chronic stress. Emergency physicians represent an important part of the airborne rescue service. Together with the pilot and a supporting helicopter emergency medical service (HEMS) employee, they constitute the crew of a rescue helicopter which is called to the scene of an emergency from a rescue station. On-scene, the emergency physicians are responsible for the primary care of the patient and accompany the patient to the nearest hospital. In order to become emergency physicians in the airborne rescue service in Germany and Switzerland, physicians need to hold a qualification according to the respective national law as well as a specialist qualification in an intensive care field (e.g. surgery or anaesthesiology). In addition, most airborne emergency physicians have a qualification in intensive care transport as well as professional experience in ground-based rescue services. Just like police officers, rescue workers, such as emergency physicians, have a particular work-related stress burden compared to occupational groups with less demanding jobs. This is characterized by serious job-related physical and mental health problems and sleeploss (Johnson et al., 2005; Hegg-Deloye et al., 2014). The medical profession, in particular, shows significantly higher burnout rates compared to other occupational groups (Lu, Dresden, McCloskey, Branzetti, & Gisondi, 2015), whereby emergency physicians have the highest prevalence with burnout rates between 50 and 60% (Lu, Dresden, McCloskey, Branzetti, & Gisondi, 2015; Estryn-Behar et al., 2011). The reasons for this heavy manifestation include confrontation with critical events (Alexander, & Klein, 2001) as well as work-organisational factors, such as shift-work and having to be on call any time, day or night (Harrington, 2001).

Since chronic stress has been proven to be associated with various mental illnesses, such as depression, burnout, and various anxiety disorders (Chrousos, 2009; Herbert, 2013; Brown, Varhese, & McEwen, 2004), the consequences are manifold, not only for emergency physicians. In addition to emergency physicians being at risk of developing serious mental or physical illnesses, patients as well may be at risk of not receiving optimal care from mentally stressed emergency physicians. What is more, sizable losses may occur to the healthcare systems due to inadequate treatment and frequent sick leave.

Despite the stressful work situation of emergency physicians, there is currently very little research concerning this specific occupational group. Initial examinations of emergency physicians deployed in the air rescue service showed that the hormonal and physiological stress levels of those affected are significantly higher on working days than on days off. (Herhaus, Schoniger, Frank, Pyrc, & Petrowski, 2017). However, the elevated cortisol and heart rate values were not reflected in the subjective stress sensation.

Until now, the effect of stressors, such as shifts and physically and psychologically demanding situations on the physical and psychological health of emergency physicians is not yet understood. Therefore, the interaction between hormonal stress levels and subjectively perceived stress levels must be examined more closely. This is of the utmost necessity to the development of both prevention and intervention measures. Since chronic stress can lead to a permanent change in the cortisol reaction (Schulz, Kirschbaum, Prüßner & Hellkammer, 1998; Fries et al., 2009), an association between retrospective (long-term) stress reports and an acute measurement of hormonal stress is possible. Initial studies show that facets of the subjectively perceived stress level can provide information on the hormonal stress level (Wüst et al., 2000; Vasiliadis, Forget, & Préville, 2012). Furthermore, a self-reported high strain on the job is associated with a greater cortisol release in the morning (Steptoe, Cropley, Griffith, & Kirschbaum, 2000). However, a connection between self-reported emotional stress measures and hormonal stress measures after psychosocial stress induction could only be found in 25% of a total of 49 studies (Campbell, & Ehlert, 2012). So far, there have only been studies on the general population or other specific groups. There are no studies that link subjectively perceived stress and hormonal stress in emergency physicians, although knowledge about a possible connection would be essential for combating stress and stress-related diseases in this endangered group: If hormonal and subjectively perceived stress were to coincide in emergency physicians, it would be easier to identify particularly vulnerable emergency physicians on the basis of subjective interviews. Therefore, we hypothesized that subjectively perceived chronic and current stress would show a positive connection to the hormonal stress level on the flight recue days, clinic days, and days off of emergency physicians (H1). Furthermore, there has been evidence that the number of years of service in the healthcare profession is positively associated with symptoms of posttraumatic stress disorder and inefficient coping approaches (Nydegger, Nydegger, & Basile, 2011). Therefore, we expected that the hormonal stress level of emergency physicians could be predicted by chronic and current stress as well as by the number of professional years (H2).

## Methods

### Sample

The study participants were recruited at two locations. To begin with, all full-time emergency physicians of the German air rescue in Dresden (helicopter Christoph 38; *N* = 22) were contacted and informed about the planned study between June and December 2015. This resulted in a sample of *N* = 20 participating emergency physicians. *N* = 2 emergency physicians did not participate because they had no working shift in the considered time period. In addition, the emergency physicians of a rescue station of the Swiss air rescue “Rega” (*N* = 20) were contacted. Again, *N* = 20 subjects (all of the contacted emergency physicians) could be committed to the study. The data were collected between June 2017 and May 2018. Of the total of *N* = 40 participants, *n* = 28 were male and *n* = 12 were female. The measurements were carried out on two air rescue days, clinic days, and days off. The duplicate measurements were treated as dependent cases, giving a total number of cases of *N* = 80. At both locations, the participants were given detailed information both orally and in writing, and informed consent was obtained. The investigation was carried out in accordance with the Helsinki Declaration. There was a positive ethics vote by the Medical Faculty of the Dresden University of Technology (EK348092011).

### Experimental Procedure

To test the hypotheses, the participants were subjected to various measurements. In order to avoid distortions, it was expressly ensured that the examination procedure was run in exactly the same manner at both locations.

For testing, 2 days on which the emergency physicians were assigned to the air rescue service (flight rescue day), two normal hospital working days (ground-based), and two days off (control day) were chosen, respectively. Before the measurement, the participants were shown how to collect a salivary sample and were additionally given a leaflet instructing them to repeat the procedure directly before the measurement. On the measurement days, the cortisol awakening response (CAR) was recorded by the participants themselves. In each case, three saliva samples were taken, the first one immediately after waking up, another one 15 min later, and the third one after 30 min. In addition, at 7:00 am, the participants applied an HRV chest strap (BioHarness™ 3, Zephyr technology, USA) that recorded their heart rate variability for 24 h, whose results were however not included in this study. In addition, the emergency physicians filled out a daily protocol on the measurement days by documenting the exact times of operating procedures or other activities as well as the perceived stress levels during these activities. Finally, the participants worked on a questionnaire package about subjective stress levels, which was collected from the participants with the other investigation material after all the measurements were completed.

### Cortisol Measurement

The steroid hormone cortisol is an established biomarker of psychological stress (Hellhammer, Wüst & Kudielka, 2009). Using this hormone, the activity of the hypothalamic-pituitary-adrenocortical axis (HPA axis) can be measured. A typical course of the cortisol concentration begins with an increase within the first 60 min of awakening. Cortisol levels gradually decrease during the day (IBL International, 2019). Possible methods for raising the cortisol concentration are with blood, urine, saliva, or hair samples. In this study, the cortisol was to be measured by saliva sample. For this, Salivetten® (IBL International, 2019) were used. For a few seconds, the subjects insert a cotton roll into the mouth so that the saliva can be absorbed. Subsequently, the saliva samples are cooled for evaluation. To check the exact measurement times, MemsCaps, which serve as storage for the cotton roles before use, are employed. These record the time each time they are opened, allowing for an accurate determination of the time intervals between samples. The saliva samples are evaluated by luminescence immunoassay. Due to the characteristic increase in morning cortisol, the cortisol awakening response (CAR) is often used to quantify the cortisol concentration (Fries et al., 2009). For this purpose, several saliva samples are taken immediately after awakening (in this study at the times + 0, + 15, + 30 min). The samples are statistically evaluated using the Area under the Curve in Respect to Ground (AUC_G_) and Area under the Curve in Respect to Increase (AUC_I_; Pruessner, Kirschbaum, Meinlschmid, & Hellhammer, 2003). These statistical parameters represent the absolute extent of cortisol change in the morning and also the increase over the 30-min period. AUC_G_ and AUC_I_ provide well-established and meaningful data to research allowing for the psychological stress levels of subjects to be measured and compared (Pruessner et al., 2003).

### Questionnaires

To record the subjective stress levels, the Trier Inventory for chronic stress (TICS, Schulz, Schlotz, & Becker, 2004) was used as a long-term measure of subjectively perceived stress (previous 3 months), and the Perceived Stress Scale (PSS; Cohen, Kamarck, & Mermelstein, 1983) was used as a short-term measure for perceived stress (previous 2 weeks). The Trier Inventory for Chronic Stress (TICS; Schulz et al., 2004) measures chronic stress levels in everyday life using 57 items (five-level response scale) grouped into nine scales. Thereby, it allows an estimation of an individual’s affective and cognitive stress response profile. It also includes a general screening scale for global stress assessment. Reliability and validity are examined extensively by Schulz and Schlotz (1999) and reach values for Cronbach’s *α* = 0.76 and 0.91.

The Perceived Stress Scale (PSS; Cohen et al., 1983) was designed to measure a person’s perceived current stress level regarding the previous month using 14 items (five-step response format). The PSS is a reliable diagnostic tool for the stress load. The scale’s reliability and validity were tested in several studies and proved to be good for the long-standing version, Cronbach’s *α* achieves *α* > 0.70 in eleven of twelve studies (Lee, 2012).

### Statistical Evaluation

The evaluation of the data was carried out with the statistical software IBM SPSS Statistics Version 25.0. First, the data were processed by identifying outliers (Senthamarai Kannan, Manoj, & Arumugam, 2015). The cortisol measurements of *n* = 3 subjects could not be evaluated and were therefore excluded from the analysis. Furthermore, the data were cleaned for missing values (less than 5% of the data), and no major deviations from the normal distribution could be observed for all relevant variables (Shapiro Wilk test supplemented by histograms; Ghasemi, & Zahediasl, 2012).

In preparation for the data evaluation, the parameters Area under the Curve with Respect to Ground (AUC_G_) and Area under the Curve with Respect to Increase (AUC_I_) were formed from the individual cortisol measurements for each measurement day (Pruessner et al., 2003).

Before the analysis, it was checked whether men and women differed in their hormonal stress level to examine whether a difference needed to be taken into account when interpreting the results.

To determine how hormonal stress is related to psychological parameters, such as perceived stress or subjective perception of distressing situations, Pearson correlations were determined between the individual AUC variables and the collected questionnaires (H1). Subsequently, step-by-step regression was used to investigate which variables would be most suitable for predicting hormonal stress on the three measurement days (H2).

## Results

### Descriptive Statistics

The total sample in this study included *N* = 40 subjects (*n* = 28 men, *n* = 12 women). Table 1 gives a closer description of the sample.

**Table 1:** Description of the sample composition

	Sample *N* = 40
Sex	
Male	28 (70%)
Female	12 (30%)
Age range	31–56
Age	40.68 (SD = 6.49)
Professional years	12.28 (SD = 8.02)
Marital status	
Single	14 (35.0%)
Married	24 (60.0%)
Separated living	0 (0.0%)
Divorced	1 (2.5%)
Second time married	1 (2.5%)

**Fig. 1:**
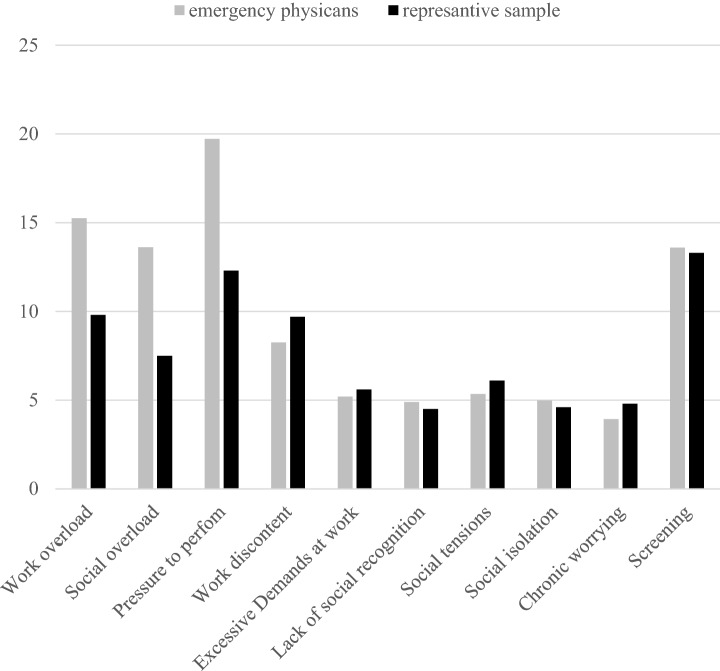
Representation of the mean values of the scales of the TICS in comparison to a representative norm sample (Petrowski et al., 2012)

The results of the individual questionnaires are shown in Table 2. It is striking that the averages of the scales of work overload, social overload, and pressure to perform of the TICS are noticeably higher than the comparative values of a representative standard sample consisting of *N* = 2,339 healthy participants aged 18–99 (Petrowski, Paul, Albani, & Brähler, 2012). Figure 1 shows this discrepancy, graphically. The results of the PSS showed no significant deviations from representative comparative samples (Klein et al., 2016; Cohen, Kamarck, & Mermelstein, 1983). The absolute measure of the cortisol awakening response of the emergency physicians and the increase in cortisol concentration are shown in Table 3.

**Table 2:** Results of the questionnaires Trier Inventory for chronic stress (TICS) and Perceived Stress Scale (PSS)

	*M*	*SD*	Min	Max
TICS				
Work overload	15.25	6.76	7.00	28.00
Social overload	13.62	3.73	6.00	22.00
Pressure to perform	19.72	5.46	6.00	33.00
Work discontent	8.25	4.21	3.00	24.00
Excessive demands at work	5.20	3.49	0.00	11.00
Lack of social recognition	4.90	3.34	0.00	11.00
Social tensions	5.35	3.70	0.00	15.00
Social isolation	4.98	3.90	0.00	17.00
Chronic worrying	3.93	2.35	0.00	8.00
Screening	13.60	7.08	2.00	28.00
PSS				
Total score	19.78	7.46	7.00	38.00

*M* mean, *SD* standard deviation, *TICS* Trier Inventory for Chronic Stress, *PSS* Perceived Stress Scale

**Table 3:** Absolute extent and increase of CAR of the emergency physicians on air rescue, clinic and free day

	Mean (SD)		
	Men	Women	Total sample
Flight rescue day			
AUC_G_	703.18 (445.28)	676.80 (348.14)	694.79 (414.32)
AUC_I_	267.25 (268.49)	192.52 (167.93)	243.47 (242.29)
Clinic day			
AUC_G_	615.14 (439.51)	576.15 (225.12)	600.52 (371.32)
AUC_I_	218.43 (172.71)	159.44 (95.20)	196.31 (150.06)
Free day			
AUC_G_	543.26 (264.90)	413.19 (172.28)	494.81 (241.30)
AUC_I_	37.54 (154.29)	80.87 (92.11)	53.68 (135.13)

*CAR* cortisol awakening response*, SD* standard deviation, *AUC*_*G*_ area under the curve with respect to the ground; *AUC*_*I*_ area under the curve with respect to increase

The extent and the increase of cortisol differed significantly between male and female subjects only in the total amount on the day off, *t*(47) = 2.123, *p* = .039, with men showing higher cortisol reactions than women.

#### H1: Association of Cortisol Awakening Response and Subjective Stress Perception

To determine the relationship between cortisol awakening response (CAR) and emotional stress, correlations were calculated between the AUC variables of the various measurement days and the questionnaires TICS and PSS. For TICS, CAR correlated significantly with some of the scales only on the day of flight rescue. The scales social overload, lack of social recognition, and chronic worries seemed to be related to both the overall CAR and the increase in CAR. The social isolation scale and screening were found to be significant only in terms of the increase in CAR. In addition, there was a significant association between dissatisfaction with work and the overall extent of CAR. Also, when looking at the results of the PSS, there were significant correlations for the flight rescue day, but not for the clinic day or the day off. In addition, it is noticeable that all significant correlations of these two inventories with CAR are negative. Therefore, H1 in particular can only be confirmed due to the negative associations between CAR and TICS and CAR and PSS as well as the insignificant results on the clinic day and the day off. Table 4 provides an overview of all calculated correlations.

**Table 4:** Results of the correlations between AUC and the psychological questionnaires

	Flight rescue day		Clinic day		Free day	
	AUC_G_	AUC_I_	AUC_G_	AUC_I_	AUC_G_	AUC_I_
TICS						
Work overload	− .060	− .148	.262	− .013	.115	− .143
Social overload	− .296*	− .267*	.003	.047	− .122	− .187
Pressure to perform	− .147	− .092	.103	.048	− .136	− .189
Work discontent	− .310*	− .225	− .251	− .163	− .123	.217
Excessive demands at work	− .138	− .222	.020	− .140	− .053	− .101
Lack of social recognition	− .275*	− .254*	.038	− .126	− .061	− .168
Social tensions	− .189	− .136	.114	.046	.128	− .039
Social isolation	− .200	− .243*	− .222	− .080	− .253	.090
Chronic worrying	− .350**	− .331**	− .083	− .200	− .269	− .131
Screening	− .216	− .299*	.101	− .099	− .061	− .191
PSS						
Total score	− .281*	− .264*	− .132	− .155	− .202	− .096

*AUC*_*G*_ area under the curve with respect to the ground, *AUC*_*I*_ area under the curve with respect to increase

*p < .05, **p < .01

#### H2: Prediction of CAR

Subsequently, stepwise regression analyses were used to examine which aspects of subjective stress perception best predict hormonal stress perception. A total of three regressions were calculated, which considered the overall extent of AUC_G_ stress on the air rescue day, clinic day, and day off. In addition to the questionnaires, the variables of the number of professional years and gender were also included in the analysis. Age was not included as it correlated strongly with years of work [*r*(72) = .851, *p* = .001] and would therefore lead to multicollinearity. The stepwise regression gives a significant model for AUC_G_ on the flight rescue day. The largest amount of variance is explained when the variables number of professional years and total PSS score are included in the model. With a corrected *R*^2^ = .335, the model has a high variance explanation (Cohen, 1988). The clinical day and day off models also achieved statistical significance. On the day off, the variables number of professional years and the PSS total also contributed to the model with the highest variance explanation. A corrected *R*^2^ = .508 was achieved. On the clinic day, the variable number of working years continued to be a significant predictor. However, instead of the overall PSS value, the TICS scales of pressure to succeed and social isolation became significant. Overall, with a corrected *R*^2^ = .599 on this day, the highest variance explanation was achieved. However, H2 could (mostly) be confirmed due to the significant influence of the number of professional years on all three measurement days and the influence of current stress on the flight rescue day and the day off as well as chronic stress on clinic days. All the results of the three stepwise regressions are shown in Table 5.

**Table 5:** Results of the stepwise regression analyses to predict the total amount of cortisol at air rescue, clinic, and free day

Measurement day	Independent variable	Standardized coefficient *b*	*F*	Corrected *R*^2^
Flight rescue day	Professional years	.523***	15.587***	.335
	PSS-total score	− .312**		
Clinic day	Professional years	.759***	20.422***	.599
	TICS-pressure to perform	.363***		
	TICS-social isolation	− .225*		
Free day	Professional years	.694***	23.239***	.508
	PSS-total score	− .237*		

*F* F-statistic; *R*^2^ explained variance, *PSS* Perceived Stress Scale, *TICS* Trier Inventory for Chronic Stress, *CISS* Coping Inventory for Stressful Situations

***p < .001, **p < .01, *p < .05

## Discussion

When the relationship between the morning cortisol response of the emergency medical physicians and the two questionnaires on stress levels was examined, some facets of stress could be related to hormonal stress levels. The currently perceived stress level seems to be related to CAR on the flight rescue day, but not to CAR on the clinic day or the day off (H1). This fact suggests that the air rescue day has a special significance for the stress burden of emergency physicians. In other studies, working days proved to be more significant with regard to the stress response than days off (Herhaus et al., 2017). Due to the negative nature of the correlations, it can be concluded that emergency physicians experiencing greater social overload, lack of social recognition, social isolation, job dissatisfaction, and chronic worries have a weaker morning cortisol response on the air rescue day. This relationship was initially in contrast to the assumption that social isolation would lead to a higher CAR (Grant, & Hamer, 2009). Nevertheless, this negative effect is not only evident in chronic stress (TICS) but could also be confirmed by the results of the measurement of current stress (PSS). The orientation of these correlations might be an indication that the situation-unspecific positive relationship between isolation and CAR reverses in extreme situations. Various approaches have shown that CAR is particularly weak in some mental disorders (PTSD or chronic fatigue; Wessa, Rohleder, Kirschbaum, & Flor, 2006; Keeshin, Strawn, Out, Granger, & Putnam, 2014; Nijof et al. 2014; Fries, Hesse, Hellhammer, & Hellhammer, 2005). Emergency physicians claiming to suffer from chronic worries, lack of recognition, and social isolation may show a permanent change in CAR due to frequent emotionally extreme situations, just like traumatized patients. Further, this raises the question, why this connection occurs only on the air rescue day and not on the clinic day. In line with the present results, a negative correlation between CAR and subjective stress (also using PSS) was found in healthy men during a final examination (Duan et al., 2013). Despite an increase in perceived stress due to an exam, these individuals showed decreased CAR values. The negative relationship between CAR and subjective stress was strongest among those participants who reported a particularly high level of subjective stress. A negative association between CAR and perceived stress was also observed a day prior as well as with physical symptoms on the day of the exam (Gartland, O’Connor, Lawton, & Bristow, 2014). Therefore, CAR is a suitable marker for chronic stress (Fries et al., 2009; Wüst et al., 2000; Inslicht et al., 2012), yet, it is also influenced by other variables (Wüst et al., 2000; Kunz-Ebrecht, Kirschbaum, Marmot, & Steptoe, 2004; Waye, Clow, Edwards, Hucklebridge, & Rylander, 2003). In specific gender, socio-economic status (Kunz-Ebrecht et al., 2004) and perceived quality of sleep (Waye et al., 2003) showed an influence on CAR in both men and women. However, the gender distribution was similar on all days and therefore did not influence our results. Due to the fact that all subjects were emergency physicians, a similar socio-economic status may also be assumed. Finally, to control for sleep quality, the shift durations of the emergency physicians were standardized, although individual differences in sleep quality cannot be ruled out. However, it must be taken into account that there are many other factors, such as individual professional experiences (e.g., physical or verbal violence) influencing CAR and general stress perception (Sun et al., 2017).

It is also striking that compared to a representative sample (Petrowski et al., 2012), the total sample examined shows noticeably higher values on work overload, social isolation, and pressure to perform. The considered sample, therefore, seems to be more heavily burdened than the general population. These findings are in line with the findings by Lu, Dresden, McCloskey, Branzetti, & Gisondi (2015) and Estryn-Behar et al., (2011) who postulate a more pronounced overall stress burden among rescue workers compared to other occupational groups.

The results of the stepwise regressions show that the number of professional years have an influence on the AUC_G_ of the measurement days. In addition, the subjectively perceived stress load seems to be important for hormonal stress processing, at least on air rescue days and possibly on days off (*p* < .05). On clinic days, though, the scales of pressure to succeed and social isolation proved to be significant (H2). The fact that professional years are positively related to AUC_G_ on all three days might be an indication that emergency physicians, who have been working in the service for many years, are more heavily involved emotionally in the rescue service than emergency physicians, who have spent fewer years working in the rescue service. This finding could be explained using the building block theory (Schauer et al., 2003). Accordingly, stressful events accumulate over the lifespan. Single loads can be processed. However, if a certain threshold sword is exceeded by repeated stress-inducing events, this accumulated load may cause a person to develop mental symptoms. As the profession of rescue services automatically leads to regular confrontation with psychologically and physically stressful situations (Alexander, & Klein, 2001; Harrington, 2001), an accumulation of these burdens over the years is conceivable, eventually leading to a stronger stress reaction in older emergency physicians.

Furthermore, the findings of the regression analysis confirm that hormonal and subjective stress levels cannot be considered as independent of each other. However, at this point, the clinic day differs from the other measurement days. For CAR, social isolation seems to be relevant on days in the clinic. The negative orientation of the regression coefficient suggests that only well-integrated rescue teams show stronger hormonal stress reactions. Perhaps they feel under pressure to maintain their position in the group through a particularly good performance. This would also explain why pressure to perform is also important only on the clinic day and not on the day of flight rescue. In air rescue, emergency physicians work primarily alone or in very small teams, whereas in the clinic, many more people are involved. It may therefore be a social phenomenon. However, it must be taken into account that this effect is only based on a significance level of *p* < .05. Since the method of the stepwise regression tends to overestimate effect, those with a significance level of *p* < .05 ought to be interpreted with caution. Therefore, this result can only be seen as an indication and not as a verified fact.

In order to rule out a possible distortion by marital status regarding the impact of social isolation on the stress level, analyses of variance were used to check whether there was a difference between marital status in terms of social isolation or cortisol. Since all calculated results were clearly insignificant, a distortion caused by marital status can be excluded.

The fact that there is only a cortisol difference between men and women in the total amount on the control day (day off) is surprising, since many present studies revealed that women show higher CAR than men (Kudielka, Buske-Kirschbaum, Hellhammer, & Kirschbaum, 2004; Maina, Palmas, & Filon, 2008). Yet, there is also first evidence that stress has a stronger impact on men than on women (Lovallo, Farag, & Vincent, 2010). Nevertheless, this single result needs to be interpreted with caution, since it could only be found in the total amount on the control day, where a possible distortion caused by different awakening times cannot be ruled out (see limitations). Further investigations are necessary to examine possible moderating factors which modify the familiar effects.

When interpreting the results, it must be taken into account that the sample with *N* = 80 cases has a relatively small scope. This is due to the narrow inclusion criteria of the study. The sample consisted only of subjects who worked as emergency physicians at the time of the survey and were assigned to the air rescue service. It is also a paired sample. The *N* = 80 cases are therefore based on a sample of *N* = 40 subjects. Another limitation is the fact that the subjects rose significantly later on their day off than on the working days (mean difference = 109 min), *F*(2, 47) = 66.897, *p* < .001. As a result, the comparability of cortisol levels on the day off compared to the working days may be limited. The analysis of cortisol levels on the day off should therefore pay particular attention to the AUC_I_ value as it only reflects the increase in cortisol and is not influenced by any change in the baseline value as a function of time. Therefore, the difference in cortisol levels between male and female participants on the control day can be neglected. This difference could not be considered for any other cortisol variable. Although the results of the stepwise regression also refer to total cortisol, the influence of the number of professional years is also found on the other days of the measurement. Further studies should ensure that the subjects rise at the same time on all three measurement days. In this investigation, rising earlier on the control day was requested but not obligatory as it might have led to the non-participation by some of the subjects and thus to the reduction of the already very specific and small sample. Furthermore, the reported results ought to be attributed only to the sample considered and be extrapolated to other samples only with caution. To extend the generality of the results, the study should be replicated employing larger samples. Finally, when interpreting the results of the stepwise regression, it needs to be taken into account that using this statistical method the effects may be overestimated. Therefore, the effects that appear only on the significance level of *p* < .05, must be interpreted with caution.

Summing up, hormonal and subjective stress may not have a unidirectional connection but may be in a more complex relationship. In order to investigate these compounds more closely and to understand the relationship between the effects, further influencing factors should be included in future research. For example, it might be examined to what extent variables such as job satisfaction or availability of extensive specialist knowledge moderate the relationship between hormonal and subjectively perceived stress levels. Building on the results, prevention and intervention strategies should be developed that reduce both the hormonal and the perceived stress levels of emergency physicians, thus reducing stress-related sequelae.

## Data Availability

The data that support the findings of this study are available on request from the corresponding author, D. Braun.
